# Knockout of PKC θ gene attenuates oleic acid-induced acute lung injury via reduction of inflammation and oxidative stress

**DOI:** 10.22038/ijbms.2021.56908.12695

**Published:** 2021-07

**Authors:** Wei Li, Xue Zhao, Ting-Ting Yu, Wei Hao, Guo-Guang Wang

**Affiliations:** 1 Department of Pathophysiology, Wannan Medical College, Wuhu, China; 2 Experimental Center for Function Subjects, Wannan Medical College, Wuhu, China

**Keywords:** Acute lung injury, Gene knockout, Inflammation, Oleic acid, Oxidative stress, Protein kinase C θ

## Abstract

**Objective(s):**

Acute respiratory distress syndrome resulting from acute lung injury has become a momentous clinical concern because of high morbidity and mortality in discharged patients with pulmonary and nonpulmonary diseases. This study aimed to explore the effect of protein kinase C (PKC) θ gene knockout on acute lung injury.

**Materials and Methods:**

Wt and PKC θ gene knockout mice were intravenously injected with oleic acid to induce acute lung injury. Pulmonary capillary permeability was assessed via measuring lung wet/dry weight ratio and level of protein in bronchoalveolar lavage fluid (BALF). Histological changes were used to examine acute lung injury. Malondialdehyde (MDA) level, superoxide dismutase (SOD) activity in serum, together with inflammatory cytokines including interleukin (IL)-6 and tumor necrosis factor-alpha (TNF-α), were determined. Furthermore, the expressions of heme oxygenase (HO)-1, nuclear factor kappa B (NF κB), and inhibitor of NF-κB alpha (IκB α) were detected in the lungs.

**Results:**

PKC θ gene knockout decreased lung wet/dry weight ratio, reduced levels of MDA, IL-6, and TNF-α in serum together with level of protein in BALF. Furthermore, PKC θ gene knockout increased the activities of SOD. Knockout of PKC θ was also observed to increase expression of HO-1 and reduce levels of p-NF κB and p-IKB α in the lungs.

**Conclusion:**

These results suggest that PKC θ gene knockout attenuates oleic acid-induced acute lung injury via improving oxidative stress and inflammation.

## Introduction

The lung is considered a primary target of various insults such as noxious gases, contaminants, and infection, subsequently resulting in lung injury. Diffuse inflammation is implicated in acute lung injury which exhibits various features such as pulmonary edema and bilateral pulmonary infiltrates (1, 2). Acute lung injury (ALI) causes proteinaceous alveolar exudates and further progresses to its severe form called acute respiratory distress syndrome (ARDS) (2). ARDS has become a momentous clinical concern because of high morbidity and mortality in discharged patients with pulmonary and nonpulmonary diseases (3). Clinical and experimental results indicate that oxidative stress and inflammation are involved in the pathogenesis of ALI (4-6). Excessive generation of reactive oxygen species (ROS) in the state of oxidative stress impairs biological membranes through lipid peroxidation, which increases vascular permeability and further results in leakage of serum proteins into the alveoli (7). Various studies have demonstrated that ROS stimulates activation of innate immune cells and the subsequent release of inflammatory cytokines such as tumor necrosis factor (TNF)-ɑ and interleukin 6 (IL)-6, with evidence suggesting that ROS, together with inflammatory cytokines, can cause vascular endothelial dysfunction and further accelerate the progression of ALI including ARDS (8-10).

Protein kinase C isozymes are a family of serine-threonine kinases which play many important roles in various physiological processes, such as cell differentiation and proliferation, regulation of gene expression, modulation of ion channels, angiogenesis, contractility of vascular smooth muscle cells and extracellular matrix proteins (11, 12). It has been reported that ROS can stimulate the activation of PKCs (13). Meanwhile, activated PKC further aggravates ROS production through increasing NADPH-oxidases (NOX) activity (14). PKC θ, a member of the PKC family, is a pivotal mediator of T-cell receptor signaling and T-cell activation (15). PKC θ is also expressed in other types of cells, such as skeletal muscle and platelets, and plays a vital role in various physiological and pathophysiological processes (16)a novel member of the protein kinase C (PKC. PKC θ is confirmed to be involved in modulation of nuclear factor kappa beta (NF-κB) via translocation to the immunological synapses (17). Furthermore, increasing evidence suggests that PKC θ deficiency enhances insulin resistance, attenuates antigen-induced arthritis, and improves muscular dystrophy (18, 19). In addition, PKC θ knockout was observed to attenuate airway inflammation in the lung (20).

Oleic acid-induced lung injury is commonly used in the study of experimental ARDS. The oleic acid-induced ARDS exhibits a similar ALI to that caused by fat embolism in patients of orthopedic surgeries, which increases the mortality of patients (21). There are many similar pathological changes between ARDS and oleic acid-induced lung injuries, such as interstitial and intra-alveolar edema, hemorrhage, and intravascular coagulation (22). An increase in the number and activity of neutrophils and macrophages initiates oleic acid-induced lung injury through increasing the production of free oxygen radicals and subsequent injury of endothelial cells and alveolar epithelial cells (23). Therefore, this study aimed to explore the effects of PKC θ gene knockout on an oleic acid-induced injury.

## Materials and Methods

***Materials***

Oleic acid was obtained from Sigma-Aldrich Corporation (St Louis, MO, USA). Specific ELISA kits for the determination of TNF-α and IL-6 were obtained from Hefei Bomei Biotechnology CO., LTD, (Hefei, China). Kits for determination of SOD and MDA were provided by Nanjing Jiancheng Bioengineering Institute (Nanjing, China). Antibodies β-actin, PKC θ, HO-1, IKB α, p-IKB α, NF-κB, and p-NF-κB were purchased from Bio Basic Inc. (Canada).

***Animals***

C57BL/6 mice (6–8 weeks old) were purchased from Changsha Tianqin Biotechnology Co., Ltd (Changsha, China). PKC θ knockout mice (6–8 weeks old) were generated on a C57BL/6 background and obtained from Shanghai Genechem Co., LTD (Shanghai, China). All animals were bred in our animal laboratory at 22±2 °C room temperature and a 12-hour light/dark alternate. Experimental procedures were approved by the Academic Experimental Animal Care and Use Committee of Wannan Medical College and in accordance with Chinese Community Guidelines for the use of Experimental Animals.

***Induction of acute lung injury***

After 2 weeks of acclimatization, the mice were weighed and received an intravenous injection of 0.1 ml/Kg oleic acid using sterile syringes to be free from bacterial contamination. Four hours after administration of oleic acid, mice were anesthetized with sodium pentobarbital (50 mg/kg), and fasting blood samples were collected for biochemical analysis. 

***Lung wet/dry weight ratio***

Following sacrifice, the mice chests were cut, and bilateral lungs were exposed. Lung tissues were separated from the surrounding tissues and excised. After removing blood from the surface, the lung tissues were weighed and placed in an electrothermal oven to dry at 60 °C for 72 hr. Dried lungs were weighed for calculation of the lung wet/dry weight ratio.

***Bronchoalveolar lavage fluid (BALF)***

At the end of the experiment, the mice were anesthetized by an intraperitoneal injection of sodium pentobarbital (50 mg/kg). A small-caliber cannula was inserted into the trachea. The lungs were washed three times with 0.5 mL of PBS to collect BALF. Lymphocytes and neutrophils in the BALF were counted by an automatic blood cell analyzer. The BALF samples were centrifuged at 1000 g, 4 °C. Protein in the supernatant was determined with assay kits (Jiancheng Bioengineering Institute, Nanjing, China).

***Determination of inflammatory cytokines***

Levels of TNF-α and IL-6 in serum were measured by commercial specific ELISA kits according to the manufacturer’s instructions. 

***Assessment of anti-oxidants***

To assess the change of anti-oxidants, malondialdehyde (MDA) level and superoxide dismutase (SOD) activity were determined in serum using assay kits from Nanjing Jiancheng Bioengineering Institute (Nanjing, China).

***Analysis of histology***

At the end of the experiment, the lungs were harvested and fixed in 10% neutral formalin for 24 hr. Subsequently, fixed tissues were dehydrated with different concentrations of ethanol in turn. After embedding in paraffin wax, tissues were cut into 5 μm sections for hematoxylin-eosin staining. Morphometric changes were observed under a light microscope.

***Western blot***

Lung tissues were separated and lysed in ice-cooled lysis buffer (50 mmol/l Tris, 1 mmol/l sodium pyrophosphate, 0.1% SDS, 1% Triton X-100, 0.02% sodium azide, 150 mmol/l sodium chloride, 0.05% Sodium deoxycholate, 2 mmol/l phenylmethanesulfonyl fluoride). The proteins were obtained by centrifugation at 12000 g for 20 min at 4 °C. The proteins in the supernatants were electrophoretically separated by a sodium dodecyl sulphate polyacrylamide gel electrophoresis (SDS-PAGE), followed by transferring to nitrocellulose membranes. The membranes were put into 5% skimmed milk containing a primary antibody overnight at 4 °C. After rinsing with PBS, the membranes were incubated with a peroxidase-conjugated secondary antibody. Antigens were visualized by DAB staining (Bio Basic Inc., Canada).

***Statistical analysis***

The data are expressed as means ± SD. Statistical analysis was performed using an unpaired Student’s t-test or one-way analysis of variance (ANOVA) and corrected using a Bonferroni/Dunn test. *P*<0.05 was considered statistically significant. Analysis was performed using SPSS v 18.0 software (SPSS Inc., Chicago, IL, USA).

## Results

***Effects of PKC*** ***θ*** ***knockout on the lung injury induced by oleic acid***

**Figure 1:**
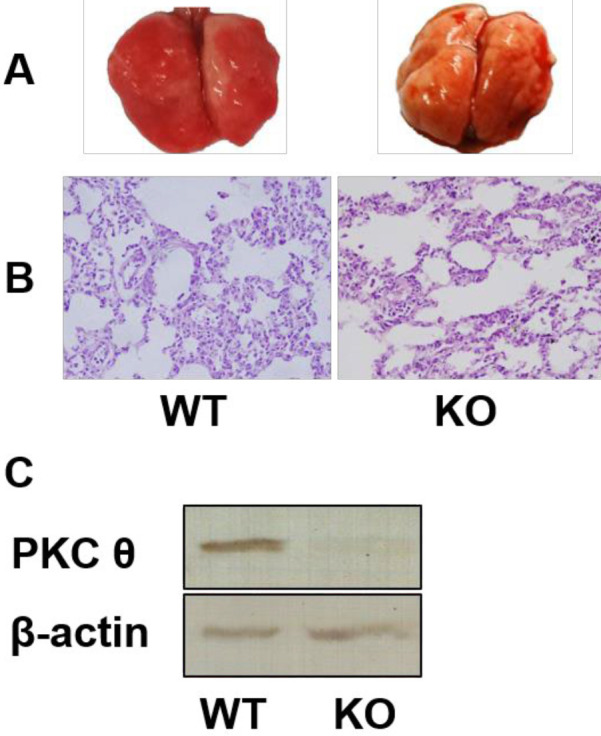
Effect of PKC-θ knockout on oleic acid-induced lung injury. (A) Feature of lung injury. (B) HE staining of lung tissues, Magnification is 400×. (C) Western blotting analysis of PKC θ

To investigate the functional role of PKC θ in the lung, PKC θ knockout mice were used to induce lung injury. Western blotting analysis showed no expression of PKC θ in the knockout mice (Figure 1C). Further, we explored the effects of PKC θ knockout on ALI induced by oleic acid. The results show that the lung in WT mice exhibits a larger area of bleeding and darker bleeding spots than those in PKC θ knockout mice (Figure 1A). In addition, there is more sputum with red blood cells in the trachea of WT mice than that in PKC θ knockout mice (Figure 1A). Histological observation showed that PKC θ knockout had alleviated oleic acid-induced pulmonary interstitial edema and reduced infiltration of cell exudation in the interstitium and alveolar spaces when compared with the WT mice (Figure 1B).

***Effects of PKC θ knockout on pulmonary capillary permeability***

**Figure 2:**
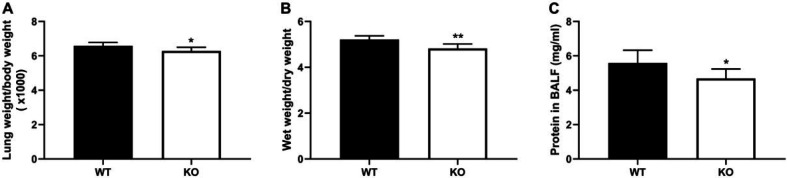
Effect of PKC-θ knockout on pulmonary capillary permeability. (A) lung weight/body weight ratio. (B) wet/dry lung weight ratio. (C) Levels of protein in BALF. *P-value <0.05, **P-value<0.01 compared with WT mice

Pulmonary capillary permeability plays a vital role in ALI (24). Therefore, one of its hallmarks, the lung wet/dry weight ratio, was determined. The results suggested that PKC θ knockout reduced lung weight/body weight ratio (Figure 2A) and led to a decrease in the lung wet/dry weight ratio compared with the WT mice (Figure 2B). Furthermore, the decreased protein concentrations in the BAL fluid were observed in the PKC θ knockout mice compared with the WT mice (Figure 2C).

***PKC*** ***θ*** ***knockout reduces lung inflammation induced by oleic acid***

**Figure 3:**
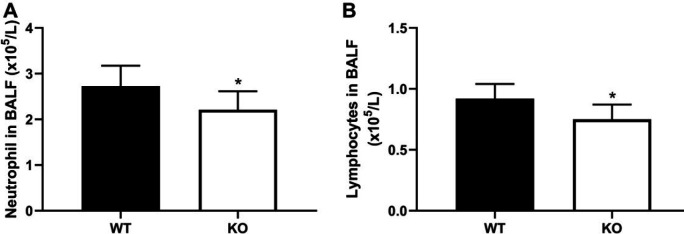
Effect of PKC-θ knockout on Inflammatory cells in BALF. (A) The number of neutrophils. (B) The number of lymphocytes. *P-value <0.05 compared with WT mice

**Figure 4:**
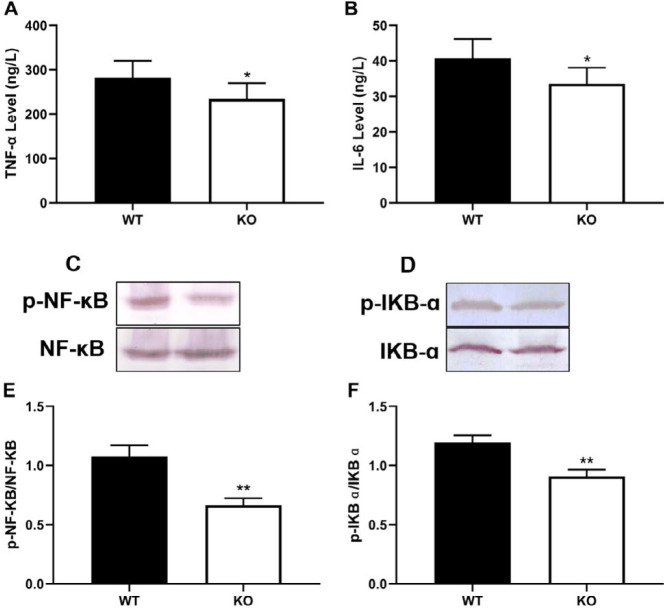
Effect of PKC-θ knockout on inflammation. (A) TNF α level in serum. (B) IL-6 level in serum. (C) Expression of p-NF κB in the lung. (D) Expression of p-IκB α in the lung. (E) Relative level of p-NF κB. (F) Relative level of p-IκB α. *P-value <0.05, **P-value <0.01 compared with WT mice

PKC θ knockout significantly decreased the numbers of neutrophils (Figure 3A) and lymphocytes (Figure 3B) in the BAL fluid compared with the WT mice. In addition, PKC θ knockout reduced infiltration of neutrophils in the lung tissues (Figure 1B). Pathological changes of ARDS are associated with local and systemic inflammation. In this study, inflammation was assessed via measuring the levels of TNF-α and IL-6 in serum. PKC θ knockout significantly reduced the levels of TNF-α and IL-6 in serum (Figures 4A and 4B).

***Reduction of oleic acid-induced oxidative stress***

**Figure 5:**
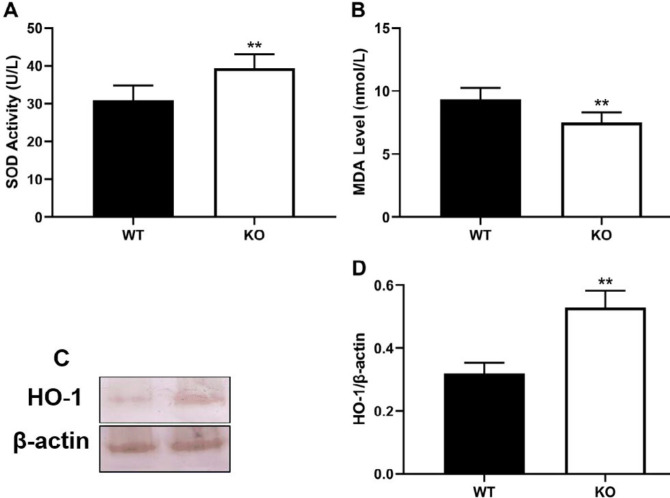
Effect of PKC-θ knockout on oxidative stress. (A) SOD activity in serum. (B) MDA level in serum. (C) Expression of HO-1 in the lung. (D) Relative level of HO-1. *P-value <0.05, **P-value <0.01 compared with WT mice

SOD: superoxide dismutase; MDA: Malondialdehyde

It is well known that free oxygen radicals are implicated in oleic acid-induced lung injury. Thus, we determined levels of SOD and MDA to evaluate the change of oxidative stress in this study. Our results indicate that PKC θ knockout significantly increased activity of SOD (Figure 5A) and decreased the MDA level (Figure 5B).

***Effects of PKC θ knockout on expression of HO-1, p-NF-*** ***κB*** ***, and p-IκB ɑ***

To further elucidate the potential mechanisms of action of PKC θ knockout in ALI, levels of p-NF-κB (Figure 4C) and p-IκB ɑ (Figure 4D), and expression of HO-1 (Figure 5C) were determined using western blotting. Compared with the WT mice, PKC θ knockout significantly increased the expression of HO-1 in the lung (Figure 5D). Meanwhile, PKC θ knockout was also observed to reduce the relative levels of p-NF-κB and p-IκB ɑ (Figures 4E and 4F).

## Discussion

ARDS results from alveolar and capillary injury and then causes interstitial pulmonary edema, which leads to pulmonary failure and high mortality (25, 26). Oleic acid-induced ALI exhibits similar pathological features to ARDS (22). In this study, ALI induced by Oleic acid was used to evaluate the effects of PKC θ knockout on ARDS in mice. The results show that PKC θ knockout attenuated oleic acid-induced lung injury in mice. PKC θ knockout was observed to decrease levels of IL-6, TNF-α, and MDA. Conversely, PKC θ knockout increased the activities of SOD in serum. Furthermore, our results indicate that PKC θ knockout up-regulated HO-1 expression, and reduced levels of p-NF-κB and p-IκB ɑ in the lung.

ARDS is a severe clinical concern caused by various factors such as infection and shock. Oleic acid-induced ALI has been demonstrated to show many features similar to those in the clinical ARDS. Therefore, a model of ALI induced by oleic acid is commonly used in ARSD studies (22). ARDS is characterized by intra-alveolar edema, hemorrhage, and alveolar-capillary endothelial and epithelial destruction (27, 28). Consistent with previous studies (22, 29), our results suggest that oleic acid treatment exhibits these pathologic changes in the lungs of WT mice. However, PKC θ gene knockout was observed to relieve the alterations. Additionally, the alveolar-capillary endothelial and epithelial destruction increases pulmonary capillary permeability and plasma protein leakage. Depositions of exudative plasma proteins and cell debris on the alveolar wall lead to formation of hyaline membranes (28, 30). In the present study, PKC θ gene knockout significantly decreased the lung wet/dry weight ratio and level of protein in BALF, suggesting that PKC θ gene knockout attenuated the alveolar-capillary endothelial and epithelial injury induced by oleic acid.

Protein kinase C isozymes have been demonstrated to be implicated in various pathological processes such as inflammatory response, oxidative stress, diabetes, and thrombosis (31, 32). Functional mechanisms of PKC isoform are deduced from studies in pharmacological modulation of PKC activity (33, 34), together with gene knockout of PKCs (35, 36). PKC θ, a member of the PKC family, is implicated in the activation of various signaling cascades (37). Overexpression of PKC θ in platelets can regulate signal transduction required for platelet activation, aggregation, and hemostasis (38). A previous study showed that PKC θ can activate nuclear factor-kappa B (NF κB) and activator protein 1(AP-1), thus stimulating the generation of interleukin 2 (39). NF κB has been reported to play a vital role in the regulation of inflammation. NF-κB is activated via phosphorylation by IκB ɑ. Activated NF κB further translocates into the cell nucleus and stimulates gene expression including TNF-ɑ and IL-6 (40). Furthermore, PKC θ gene knockdown decreases levels of IFN γ, IL-6, and TNF α, and ameliorates the inflammation responsible for liver injury (41). 

An excessive inflammatory response is responsible for the pathogenesis of ARDS via alveolar edema, hemorrhage, and hyaline membrane formation (42). Some studies have demonstrated that activated macrophages, microvascular endothelial cells, and alveolar epithelial cells trigger the production and release of pro-inflammatory cytokines such as IL-6 and TNF-α, expression of adhesion molecules and generation of ROS (43, 44). Furthermore, Neutrophil migration into the lung and infiltration exaggerates inflammatory response (45). Clinical and experimental data show that oxidative stress is involved in the pathogenesis of ARDS via ROS to destruct biological membranes, increasing leakage of plasma protein into the alveoli (46). In addition, it has been reported that ROS can stimulate the activation of macrophages and neutrophils, increasing the expression of inflammatory cytokines and impairing vascular endothelial cells, which exacerbates ARDS (8, 10, 47, 48). Changes in the activities of SOD, c-Jun N-terminal kinase (JNK), and mitogen-activated protein kinase (MAPK) are involved in the regulation of inflammatory responses under oxidative stress (49). Meanwhile, several studies suggest that oxidative stress results from oxygen or inflammatory responses, and the release of inflammatory cytokines triggers oxidative stress via induction of ROS (50, 51). Heme oxygenase 1 (HO-1) has been reported to exhibit anti-oxidant and cytoprotective roles (52). HO-1 is also demonstrated to play an important role in mediation of inflammation (53), and with evidence revealing that induction of HO-1 suppresses NF-κB/IκB ɑ signaling (54). In the present study, our results show that PKC θ gene knockout decreased levels of IL-6, TNF α, and MDA in serum. In addition, PKC θ gene knockout was also observed to increase the activity of SOD, elevate expression of HO-1, and reduce levels of p-NF κB and p-IκB α. These findings suggest that PKC θ gene knockout attenuated oleic acid-induced ALI by regulating inflammation and oxidative stress.

## Conclusion

In summary, our results show that PKC θ knockout can relieve lung injury induced by oleic acid and decrease levels of hallmarks of pulmonary capillary permeability such as lung wet/dry weight ratio, number of neutrophils, and level of proteins in BALF. Furthermore, our data also revealed that PKC θ knockout improved oxidative stress and inflammation via elevating the expression of HO-1 and down-regulating the NF κB/IκB α pathway. These findings suggest that PKC θ can be a therapeutic target for lung injury.
